# Workplace Violence Against Nurses Working In Mental Hospitals In Oman: a cross-sectional study

**DOI:** 10.1192/j.eurpsy.2023.1831

**Published:** 2023-07-19

**Authors:** M. S. Alkalbani

**Affiliations:** Psychiatry, Oman Medical Specialty Board, Muscat, Oman

## Abstract

**Introduction:**

Workplace violence (WPV)has been a persistent problem that is underestimated and generally disregarded by the public and professional organisations, and it has been noticed that the highest number of these assaults are directed towards healthcare workers.

**Objectives:**

The aim of the study is to determine the prevalence of workplace violence against nurses in psychiatry hospitals in Oman and to explore the determinants contributing to the workplace violence

**Methods:**

This is a cross-sectional study was conducted at two tertiary mental health care hospitals (Al Masarra Hospital and Sultan Qaboos University Hospital) in Muscat, Oman between October, and December of 2021.Participants were asked to complete the Workplace Violence in the Health Sector to assess level of violence, as well as a sociodemographic survey.

**Results:**

This study included 106 participants with 80% response rate. The Majority of participants aged between 30-39 years. Of this study, 52.8% werefemale and most of the participates are Omani (73%) and working in inpatient (80%). The highest type of violence experiences was verbal violence 86.8%followed by physical violence 57.5%. Most physical violence incidents 26.4% took place during weekdays with highest incidences happened during morning shifts (34%) followed by afternoon shift (25.5%).

**Conclusions:**

Current study revealed a relatively high prevalence of WPV among nurses working at psychiatric hospitals in Oman. Future studies needed to explore the predictors of violent among nurse. It is important to invest in the prevention of WPV by constant training of workers in mental hospital in Oman on how to respond to violent psychiatric patients.

**Disclosure of Interest:**

None Declared

